# Modus vivendi, Toleration and Power Modus vivendi, Toleration and Power

**DOI:** 10.1007/s11406-016-9798-4

**Published:** 2017-04-05

**Authors:** Glen Newey

**Affiliations:** Department of Political Philosophy and Ethics, Leiden University, PO Box 9500, 2300 RA Leiden, Netherlands

**Keywords:** Toleration, Ideal, Modus vivendi, Counterfactual, Power

## Abstract

This article deals with modus vivendi, toleration and power. On the face of it toleration and modus vivendi are in tension with each other, because of the power condition on toleration: that an agent is tolerant only if they have the power to engage in an alternative, non- or intolerant form of behaviour, and this seems to be absent in modus vivendi. The article argues that the scope of the power condition is unclear, but might be thought much more extensive than usually supposed. This becomes clear when the agent’s thoughts are subjected to a counterfactual test, concerning what would occur in their ideal world. However it is in the nature of ideals that they cannot usually be subject to a counterfactual variation here, since they determine the ideal world’s content. The article concludes that only a commitment to the other party’s freedom for its own sake proves robust in the face of counterfactual idealisation, but that it is questionable whether the dispositions that characterise toleration should be subject to so demanding a test.

## Introduction

John Horton's work has encompassed a number of distinct themes. His early articles offered powerful and sensitive contributions to the analysis of toleration. Much of Horton's middle period was devoted to setting out and developing his associationist account of political obligation. Latterly his interest in what has come to be known as the debate over realism and ideal theory has led him to elaborate a *modus vivendi-*based political theory, based in part on his reading of Oakeshott et al. (2005) and Gray (2007). Throughout his work Horton has displayed acute awareness of the embodied status of political and moral actors, and a sense that the complexities of moral and political life are often belied by the abstractions of theory – an insight which, I suspect, underlies his extensive engagement with those complexities in imaginative fiction.^1^

In this paper I am mainly concerned with an apparent tension between what Horton calls, and defends as, the ‘traditional’ account of toleration,^2^ and the idea (*ideal* may be too strong a word) of *modus vivendi*. The tension, summarily put, lies between two *aperçus. Modus vivendi* seems typically to arise when two or more parties strike a compromise because none has enough power to force through its preferred regime. However, on most traditional accounts of toleration, one tolerates only if one has the power to do otherwise. Hence the quite widely-held notion that *modus vivendi* itself constitutes a form of toleration is called into question. If *modus vivendi* simply amounts to a stalemate between two sets of people, each of whom would rather they were able to impose their will on the other, it is hard to square with toleration. Each would prefer to enforce a monoculture on the other, and given this stance, neither seems to count as tolerant. Instead each puts up with the other on sufferance, for want of ability to dictate what it regards as a more congenial solution; a mere *pis aller.* 3

When we consider what is given and what is counterfactually variable in thinking about toleration, particularly as regards power, we come up against limits to a certain form of ideal theory in the face of power. The latter questions people’s ethical or other practical commitments by asking whether they would still exist in certain counterfactual situations. I will suggest that the only obvious candidate commitment that survives this counterfactual test is that of freedom, in either a Kantian or (perhaps) some other form. Toleration, on this view, is inextricably mired in the less than ideal conditions of the here-and-now. No doubt this helps to explain why so many commentators find it awkward. I will however also question whether the test must be pressed this far.

I follow an approach which is more analytical than the one Horton is inclined to favour, proceeding by way of candidate definitions, and subjecting them to critical scrutiny. But this is to some extent a heuristic device. That is because, while this approach can advance understanding, its limits become apparent precisely when trying to make the condition analytically exact.

## Power and the Power Condition

This paper has more to say about toleration than about power. That is not, clearly, because the notion of power demands no explication. I assume that power involves *capacity*: it is within an agent’s power to do something if, in the circumstances,^4^ she has the capacity to do it. So, to lack power is to suffer a lack of capacity with respect to an action. Having the capacity to do something can be understood in an occurrent or in a dispositional way. For instance a person who has the dispositional ability to perform an action, such as piloting a passenger aircraft, may find herself temporarily (i.e. occurrently) unable to do so, for example through illness or a sudden loss of resources (such as the unavailability of an airworthy plane); however, the distinction between occurrent and dispositional capacity does not much matter for present purposes. When toleration is at issue politically, the question will usually be whether to tolerate something, and that will mean that the capacities are engaged here and now. What capacities one has depends importantly on the resources at one’s command. That may in turn depend on luck. Insofar as toleration depends on capacity, then, it will depend on these other things on which capacity itself depends.

The lack of capacity suffered by a powerless agent may result either from the agent’s own inability, or from limits in the practical environment where he is situated. I currently lack the power to jump fifty feet in the air. I may acquire the power to do so if provided with appropriate mechanical aids. This shows that the level of power enjoyed by an agent can be varied, by imagining that he has capacities which he currently lacks, or conversely. Then we can ask what this person *would* do had she power, that is, a capacity, which he in fact lacks. But even if I can jump fifty feet in the air with mechanical aids it will still not be within my power to do so unaided. Some things are not within my power as things stand, while others are not within my power even dispositionally.

It is a significant feature of thinking about power that it both admits of counterfactual variation, and that the scope for this variability can be limited, for example by imposing further conditions. These conditions minimally include that of non-contradiction. In a strong sense, it is not within my power both to be here and not to be here, now. Leaving logical impossibility aside,^5^ there is still ample room to imagine that the world might be sufficiently different to give an agent powers which he currently lacks, or conversely. In some cases, further, the counterfactual names a possibility which is or was accessible via the person’s own agency. This will be a small subset of the set of counterfactual possibilities.

There is a consensus among writers on toleration that if an agent A tolerates the performance of some action P, then A has the power to prevent or otherwise sanction P.^6^ I shall refer to this as the *Power Condition* on toleration (the numeral ‘1’ indicates that further formulations will follow).

### Power Condition 1 (PC_1_)

A can only tolerate a disapproved-of action or state of affairs P if A believes that it is within his power to prevent (etc.) P.

Non-toleration may of course take the form not of outright prevention but of censure, condemnation, or imposing other sanctions on those who are disapproved of: hence the ‘etc.’ Henceforth I take this as read.

I by-pass an important recent trend in philosophical writing on toleration, itself a target of Horton's critique, which demurs at seeing toleration in terms of prevention and permission.^7^ This approach distinguishes the practice of toleration in, particularly, the early modern period, where toleration was often granted on the basis of royal prerogative, from the form which toleration should take in liberal democracies, where those subject to power are also those who ultimately wield it. This ‘respect’ conception of toleration^8^ works with a conception of public justification according to which citizens acknowledge that disapproval arising from reasonably rejectable conceptions of the good life cannot reasonably be imposed on others. Thus, for instance, even if I subscribe to a form of Christian fundamentalism which reprobates homosexuality on Biblical grounds, I acknowledge that it is unjustifiable to use the power of the state to impose my views by force of law, since my Christian outlook is one which others may reasonably reject. On this model, toleration is exercised not from the top downwards, but via a democratic political will in which power is divided equally among those subject to law.

I shall not comment in any detail here on this approach to toleration. However, the respect conception still enables us to talk of prevention and permission. It can talk of legalising narcotics, for example, where the public justification test rules that outlawing them would rest on reasonably rejectable ideas of the good. Moreover, it will still be appropriate to think in terms of the exercise of power, even on this revised view of its legitimate use. A legitimate state has the capacity to use the legal and judicial means to enforce the criminal law. To that extent the legalising of narcotics would mark the state’s eschewal of coercion. It may however be pointed out that political capacity in a democratic state is more complex than this, as it depends not simply on brute force but on the wider means available to political leaders in canvassing acceptance for public policy. This includes arguments of principle, examined further below.

Before this elaboration, however, let us first consider the intuitive basis for endorsing the Power Condition. Since PC_1_ is necessary for toleration, it will void toleration where the non-prevention of a disapproved-of action or state of affairs results solely from the agent’s lack of power to prevent it. Suppose, for instance, that a racist would gladly repatriate immigrants if he had the power to do so, but in fact lacks that power: he finds himself in a minority which cannot dictate political outcomes. Such a person could not plausibly be described as tolerant. This reveals the intuitive basis for PC_1_: I cannot be said to tolerate something which I disapprove of or dislike, if my failure to act against it results solely from the fact that I cannot do anything about it, and I would act against it if I thought it lay within my power to do so. In such circumstances, I should be said to *put up with* the activity, rather than to tolerate it.

Can I be said to tolerate an activity I dislike or disapprove of, despite lacking the power to act against it, if I would not prevent the activity, even if I (believed that I) had the power to do so? It could be argued that in this case I do tolerate the activity. If I wrongly believe that I have the power to stop the activity, but decide on principle not to, it seems that still I act tolerantly: what matters is my *stance* towards the activity, which is not to use the power I believe that I have to prevent it. But if so, it seems hard to see what difference is made if I correctly believe that I lack the power to prevent it. The stance I take is the same, a principled eschewal of the use of power. If so, one could conclude that what is necessary for toleration is not to have the power of prevention, but not to be disposed to use the power if one believes one has it. This suggests that the PC_1_ should be modified along the following lines, which adds a further necessary condition (that is, (ii)) to the one mentioned in PC_1_.

### Power Condition 2 (PC_2_)

A can only tolerate a disapproved-of action or state of affairs P if (i) A believes that it is within his power to prevent (etc.), and (ii) it is not the case that A would seek to prevent P, if A believed he had the power to do so.^9^

This is a necessary and not a sufficient condition of toleration. On any view, more conditions need to be met. Yet someone who meets PC_2_, and the supplementary conditions, may still fail to tolerate. This is because the capacities relevant to assessing toleration can in turn depend on whether a particular option is deliberable. Someone may decide that because an option incurs unacceptable costs, it cannot be taken. For instance, I could perhaps just afford (i.e. I have just enough money) to buy some luxury item, but given that no money would be left for more important purchases, I may regard the cost as unacceptable. Again, in the circumstances of toleration, the government or some other agency may decide that suppressing some group, though possible, would incur undue costs. As this example suggests, the capacities relevant to power are scalar properties, rather than applying in an all-or-nothing way. This is unsurprising, inasmuch as the resources on which capacities often depend likewise admit of degree.^10^ More significantly, when an agent fails to prevent a disapproved-of action because doing so will incur certain kinds of cost, this will void the claim that she acts tolerantly.

To see this, consider the following example. Martha has it within her power to stop her daughter Lois from going out one evening, but fails to do so. However, Martha fails to do so only because the costs of prevention are unacceptably high to her: Lois has convincingly threatened that she will hurl a chair through the window unless she gets her own way. Martha decides not to use the parental power she has to thwart Lois’s plans. Here, although Martha fails to use her power to stop Lois from going out, her failure to use her power results only from the fact that to do so would inflict unacceptable costs on her, Martha.

This does not mean that she tolerates Lois’s going out. Of course, the fact that Lois can use the threats itself restricts Martha’s power, but it does not simply prevent Martha’s prevention. Lois makes Martha’s antecedently preferred option, that Lois stays at home and the chair does not go through the window, unavailable. But, since by hypothesis

Martha could stop Lois if she were prepared to bear the cost of doing so, it is mistaken to say that she cannot prevent what she disapproves of. Martha is forced to put up with something that she finds very disagreeable, for fear of suffering something yet worse. Here again, ‘forced’ includes the inducing of an outcome by making the alternative to it unsustainably costly. So it seems that the power condition needs to be interpreted in such a way that an agent’s power can be removed or weakened by imposing costs on non-prevention which the agent, the putative tolerator, regards as unbearable.

One might think that it does not matter whether we say that Martha lacks the power of prevention here, and so doesn’t tolerate her daughter’s going out; or that she has the power, but fails to exercise it because of the costs, and so still doesn’t tolerate it. These are, to be sure, not simply two descriptions of the same situation, because on the one account Martha has the power of prevention, and on the other not. However, what makes the matter indifferent as regards toleration is that Martha’s *attitude* remains the same in each case. As Martha sees things, it would be better if she had the power to stymie her daughter’s plans without the attendant costs. As far as this goes, what matters for toleration is not so much the extent of Martha’s actual power, or even her beliefs about that power, but her attitude in response to conduct of which she disapproves.

At least two implications follow from this discussion. First, the example suggests that PC_2_ should be elaborated to respect the intuition than an agent does not tolerate a practice if the failure to prevent it is due only to the fact that prevention incurs unacceptable costs. Martha *would* prevent Lois’s going out if doing so did not incur costs which she does not wish to bear. Then the limits on her power, duly augmented to allow for the unacceptable costs of non-prevention, will negate the claim that she acts tolerantly. The situation would be different with a more benign Martha who, though she disapproves of Lois’s nocturnal excursions, nonetheless permits her to go out, not because of some threat Lois has made, but because she, Martha, subscribes to a principle of autonomy.

Second, as regards the prospective costs of prevention, it is often unclear whether a given action is tolerant. Self-restraint frequently rests on mixed motives of pragmatism and principle. So there can be reasonable uncertainty as to whether an agent restrains himself because he regards the alternative as unduly costly, or for reasons of principle. But the distinction between ‘costs’ and ‘principles’ itself is far from clear-cut. With certain considerations, it may be doubtful how far an agent mindful of them in not preventing an action of which he disapproves can be said to tolerate that action. This is particularly true in political decision-making. For example, military intervention to redress human rights abuses in contemporary Zimbabwe would run various kinds of risk*.* Reasons of principle as well as cost may deter foreign governments or the international community from military intervention. But given that governments legitimately curate the national interest, even considerations of cost might be thought a principled reason for (in) action.^11^

Certainly, a pragmatic compromise may be the best outcome available in the circumstances. But are the parties to it acting tolerantly? A policy which some might describe as ‘tolerant’ in its *effect* – which may mean simply that it involves a compromise – need not be accompanied by a tolerant *attitude* from its authors. In fact, they may be only too willing to put their adversaries to flight if granted the opportunity. Such is the stance taken by the racist discussed earlier. He would cheerfully repatriate the immigrants given the chance. But in no sense does his behaviour strike one as tolerant. In his favoured world, those who share his views would have the power to repatriate those of whom he disapproves. Hence a policy can be tolerant in its effects, even though none of the parties to it has a tolerant attitude. Where, as in *modus vivendi*, non-suppression rests purely on stalemate, the argument so far suggests that the parties to it should not be seen as tolerant.^12^

The power condition should take account of the fact that prevention, though not impossible for the agent, nonetheless incurs costs which she judges too heavy to bear. So we need to modify PC_2_ in turn, and arrive at:

### Power Condition 3 (PC_3_)

A can only tolerate a disapproved-of action or state of affairs P if (i) A believes that it is within his power to prevent (etc.), and (ii) it is not the case that A would seek to prevent P, if A believed he had the power to do so even if, in A’s judgement, prevention did not incur undue costs.

It might be thought that what underlies this newly revised version of the test is the fact that judgements of toleration are irreducibly normative – that one can judge that A acts tolerantly only if one thinks that the reasons for A’s self-restraint are *good* ones. However, this is mistaken. One may judge that someone’s self-restraint is tolerant, even if the principle on which it is based, or the belief that the principle applies in a given case, seems misguided, as with a parent whose reason for tolerating his child’s uncouth antics lies in a misguided belief in self-expression. It is for this reason that a normative conception of toleration that claims only justified disapproval, or justified responses to disapproval, can count as tolerant, does not adequately answer the censorious tolerator problem, which Horton first exposed^13^: namely, that someone who, keeping his behaviour constant, extends the range of activities of which he disapproves, and so seemingly, and counter-intuitively, becomes more tolerant by disapproving more.^14^ This criticism could perhaps be met by qualifying the normative judgement, so that it makes not an all-in assertion about justification, but claims only that what conforms to the structure of toleration is *prima facie* justifiable. Conversely, someone who restrains himself from condemning vandalism carried out by a mob of youths, because to act otherwise would risk serious injury or death, is not acting tolerantly, even though one may accept that these reasons for self-restraint are good ones. Not all good reasons ground toleration, just as toleration can be grounded on reasons that are not good.

However, PC_3_ still fails to give an adequate representation of the role of power in judgements about toleration. This is implicit in the commentary on the Zimbabwe example given earlier. For the costs themselves may be measured in terms of *principles* which A upholds. In this case it seems wrong to treat acting on reasons of cost as voiding toleration. I may be unable to bring myself to do something because of some principle I hold, rather than because of simple lack of power, or pragmatic costs. For example, I may be unable to bring myself to torture a baby, not because I lack the physical, pecuniary, etc., resources to do so, but because I simply find myself unable to do it, on grounds of repugnance. I cannot do it, and this inability results from my principled objection to torture.

It seems that this kind of inability should fall outside the scope of PC_3_. Someone who subscribes to the principle of respecting autonomy, for instance, may decide that she cannot breach the principle even though doing so will license behaviour of which she conscientiously disapproves. Such might be the case, for instance, if Martha were more liberal-minded. Clearly this will become more likely, the heavier the moral costs (as judged by the agent) incurred by breaching the principle of autonomy through coercive intervention. Here the prospective costs, reckoned in terms of autonomy, may indeed counsel self-restraint. But if A permits another person to act in a way of which A disapproves, because A subscribes to a principle of autonomy, there seems no reason not to describe her behaviour as tolerant.

Perhaps *in extremis* even principles may be idealised away. For example, I may come to think that I arrived at the principles I now hold through some cognitive malfunction or misadventure, such as brainwashing (or simply strong social conditioning) in early life.^15^ I might then think that though I couldn’t help believing in and acting on what I believe, in a better world I wouldn’t adhere to these principles. The concern is not about backsliding – whether I manage to remain true in my actions to the principles I consciously endorse – but rather about my grip on the principles themselves. I may come to think that in my ideal world, I would adhere to some different set of principles from those I actually hold. Perhaps, if a person were to exercise self-restraint because of a principle in which he believes, while holding the further belief that his belief in this principle is a regrettable aberration, it would be wrong to affirm that he acts tolerantly.^16^

Perhaps, by the same token, someone who restrains herself from acting on a principle which licenses disapproval of some practice, *does* act tolerantly. This situation is fairly common. Someone who has been brought up to believe that eating pork is bad, unclean, and so on, may come to think that these attitudes result from conditioning in early life, but lack any objective justification. Nonetheless, she may still feel residual distaste – or more than that – at eating pork. To overrule one’s own long-established attitudes of disapproval after coming to regard them as prejudices might be thought well-nigh paradigmatic of toleration. It is not obvious, at least, that someone who struggles against such attitudes of disapproval which she finds inexpugnable counts as less tolerant than someone who, never having been given the underlying prejudices, sees the practice as unexceptionable.

The idealising disowning of one’s actual attitudes can thus work in either direction: towards undermining either the principle which supports toleration, or the attitude of disapproval. There is little gain in trying to regiment phenomena which resist it, not least because conflicts of attitudes are both a psychological commonplace and hard to pin down, as the presence of each attitude constitutes evidence for the absence of the other. Judgements about toleration have to take cognizance both of agents’ actual attitudes and of their resultant ideal outcomes – including, in the range of cases just discussed, attitudes about these very attitudes.^17^

Power does play a role in judgements about toleration. But its significance lies in the underlying *attitude* of the putative tolerator, which can be brought out by examining the counterfactuals which apply. Someone who fails to exercise her power of intervention will nonetheless fail to act tolerantly if her failure results solely from an aversion to non-principled costs, though the line between principled and other costs is often difficult to draw.

## Toleration and the Limits of Counterfactuals

The counterfactual reasoning allowed for so far permits judgements about when agents act or fail to act tolerantly. The counterfactual sifts correct and mistaken judgements by asking how agents would act in similar circumstances, adjusted to take account of their relevant capacities. The guiding intuition has been that an agent who fails to exercise her capacity to prevent activity of which she disapproves can only be judged to behave tolerantly if her self-restraint rests on certain kinds of reason. Only actions duly informed by principle will be eligible for description as tolerant. The analysis thus imposes some normative constraints on that description.

However, some could say that the constraints do not go far enough. It may be argued that there has to be a non-contingent link between the principle that supports self-restraint and the fact that the action tolerated provokes disapproval. Otherwise, it seems, one can always construct counterfactual examples which pull apart the grounds for self-restraint, on the one hand, and the possibility of disapproval, on the other, and then use a counterfactual claim to show that the agent has not, after all, acted tolerantly. In the agent’s ideal world, the empirical conflict between the reasons which support prevention on the one side, and permission on the other, would not exist.

Consider, for example, civil liberties to which those suspected of or charged with criminal offences may lay claim. In liberal polities the relevant entitlements include no detention without charge, freedom from torture, due process, access to legal representation, and so on. A foreseeable result of these safeguards is that some people who are really guilty of an offence are either not charged or are acquitted at trial. To this extent, the system of legal entitlements for detainees could be thought of as tolerant. The state, as embodied in the judiciary, permits that of which it disapproves, namely the release or acquittal of those who are really guilty, in the name of a principle, or set of principles, of personal liberty. Moreover, we may assume that the state has the power to remove these entitlements, for example by primary legislation, as has in fact happened in some countries.^18^

So far, then, it would seem, so tolerant. But is it? The objector may say: to be sure, we lack a system of (in Rawls’s phrase) perfect procedural justice^19^ in all actually existing criminal jurisdictions. That is, all systems of criminal justice are fallible as methods for determining criminal guilt, and so are only imperfectly procedurally just. But we could idealise away these shortcomings by imagining a system of criminal justice in which there is a perfect correspondence between substantive guilt under the law and the verdicts reached in criminal trials. Then, the objector continues, the rationale for the safeguards, which rests on fallibility, would be removed. In this case, the liberal state’s commitment to civil liberties would be seen as contingent on the fallibility of its own legal system. In its ideal world, in which ‘Guilty’ verdicts perfectly track substantive guilt, the state would dispense with the liberties, as their rationale would have been exhausted. So the liberal state’s claim to toleration turns out to be as flimsy as Martha’s.

One response is that the rationale for civil liberties is not exhausted by the fallibility of judicial systems. However, the underlying point remains when toleration rests on contingencies that can be imagined away with augmented capacities. Then the counterfactual threatens to call into question the commitment to non-prevention itself, since whatever gives rise to disapproval can be imagined away. The basic version of this thought is that in a better world the activity disapproved of would not exist, and so no need for toleration would arise. Surely anyone who believes that the activity is a fit object of disapproval is committed to thinking that it is *pro tanto* bad – which means that it would be better that the activity did not exist.

The objector in effect says this to defenders of toleration: Imagine yourself to be omnipotent; then, if you think that the activity really merits disapproval, why would you use your power to create a world which contained that activity? Surely you wouldn’t. But if not, you are really no different from the pragmatic ‘tolerator’ who permits what she disapproves of solely because of the costs attendant on not doing so. For in each case the reasons which counsel non-prevention result merely from a deficit of power. Martha’s claims to act tolerantly were refuted by the fact that in her ideal world, she would have no need to restrain herself from preventing her daughter from going out – since in that world no costs would be incurred by prevention; or, more ideally still, her daughter would not wish to go out anyway. So, by the same token, in this world you lack the power to prevent the activity you disapprove of; but in your ideal world, you could ensure that it never occurred. In this sense, the idea that you are committed to ‘toleration’ is self-deluding.

This line of objection may be compared with critiques of toleration offered by Herbert Marcuse and Wendy Brown.^20^ These critiques, broadly speaking, level against liberalism the charge that its profession of toleration is at best compromised by its exercise of power, which differentially benefits certain groups or interests. The present objection holds, by contrast, that the appearance of toleration actually results from a *deficiency* of power on the part of the *soi-disant* tolerators. Their ideal world is one in which the activities tolerated fail to occur, and thus the call for toleration exists only where liberals’ shortage of power means that they have to put up with the activities willy-nilly, because the costs of suppression are still greater. Then the question is whether anything can rightly be labelled ‘toleration’ at all.

We began by observing that tolerating a practice seems to require having the power to act against it. But sometimes when an agent has but fails to use this power, intuition baulks at saying that she behaves tolerantly. The question immediately follows, how genuine acts of toleration are to be distinguished from mere non-prevention. The initial answer to this question drew a distinction between ‘costs’ and ‘principles’ as constraints on the agent’s powers of prevention. However, this distinction seemed ad hoc, and in need of amplification. We sought this by considering what the agent would allow, given the power to create her ideal world. And this seems to make more sense of the distinction between principles and adventitious costs. For principles are such that they are immune to counterfactual critique, since the principles themselves go to determine what the counterfactual ideal is. Costs can be counterfactually varied between this and the ideal world, but principles cannot, because they serve to frame the ideal world itself.

Consider the example of the Holocaust. During World War II, the Nazis could have diverted further resources from other fronts to track down and kill yet more Jews. The costs of detecting and apprehending remaining Jewish fugitives were presumably subject to rising marginal costs. The fact that the Nazis failed to devote more resources to genocide hardly shows that they were tolerant of the remaining Jews’ existence. We can suppose that they would have killed them had it not been deemed unacceptably costly to do so.^21^

Revert now to the case in which the principles themselves might be thought sub-ideal.^22^ One could perhaps think that the principles one upholds are simply the result of adverse conditioning, from which one might have been free in some happier alternative biography. Whether someone who fails to use her powers of prevention on the strength of a principle which she regards in this light counts as tolerant is not immediately clear. If a person acts on principle despite holding the meta-view it may still seem reasonable to describe her as tolerant, especially if her attitudes evince the struggle with conditioned responses already discussed.

While not impossible, such a case clearly does not address the much commoner situation where the principles are not so regarded. Most people do not take this meta-view of their principled beliefs. But now the challenge is to explain why, not the principles themselves, but the activities to which tolerators apply them, should not be able to be counterfactually jettisoned in the name of moral maximisation. The explanation should not be vulnerable to the charge that permission is revocable counterfactually, given the power to create an ideal world. The range of candidates for such a non-contingent basis is likely to be small. For it will have to show both why permission is a good idea even though it countenances the doing of sub-optimal acts; while also showing why it would not be better, had one the power, to bring about a world in which these acts did not occur. Since by hypothesis the acts are sub-optimal, the explanation will need to point out some morally significant concomitant of the acts which would make it better not to bring that world about.

An obvious response suggests itself. We do not, alas, live in anybody’s ideal world. That is what gives rise to the circumstances of toleration. At both the personal and political levels people are confronted with the decision whether or not to prevent disapproved-of activities. Given that this is so, and no power on earth can change it, persons and political actors have to decide what to do for the best. A theory of political toleration, in particular, should be firmly rooted in the real-world circumstances in which decisions have to be made. The demand that real-world commitments be checked against counterfactual scenarios repeats an error which ‘realist’ critics of liberalism have recently targeted.^23^

An objector may say that this response misses the point. The challenger did not ask whether prevention was a good thing in the circumstances – which must presumably be judged on a case-by-case basis. Instead, the challenge, even when permission is deemed preferable to prevention, is whether this action can be described as tolerant. And, it may be said, there is still a difference between the objectionable cases and those in which the agent acts tolerantly. With Martha, her aim is to prevent a real person, her daughter, from acting in a certain way. According to the counterfactual test, an ideal world the activity will not arise in the first place.

But this response hardly seems to dispose of the problem. The position now is that the person who tolerates a practice, rather than merely putting up with it, does so in virtue of holding to some principle: one that says that, though permission is better than prevention given that the practice is going on, it would be better still if one had the power to create a world where it didn’t go on at all. But this hardly seems typical of toleration. If anything, it seems to verge on megalomania.

However, reflecting on why this response is inadequate helps, I believe, to get clearer about the relation of toleration and power. Take a still more extreme case: suppose I imagine a world in which everyone acts as I think best, and none acts in ways of which I disapprove. One version of this idea, familiar from philosophical objections to theodicy, would be a world over which I enjoy absolute control, so that nobody gets the opportunity to incur my disapproval. But what about a second version, in which my disapproval is never provoked – not because I exercise control over people, but because each person freely chooses to act in ways of which I approve (or at least don’t disapprove)? How could anyone object to this on the grounds of *freedom*? Whether this is coherent depends on whether it is coherent to imagine a world in which everyone freely avoids doing anything I think they should not do.

The world contains bad things, some arising from human agency. According to theodicy, these bad things come about as a necessary consequence of the fact that human beings have free will. I do not aim to revisit, still less add to, these discussions. The point is that we have now arrived at a specification of the basis for non-interference, as both knowingly licensing sub-optimal acts, and not revocable in an idealised world. Earlier I noted that the range of candidates for such a non-contingent basis must be small. In fact, it seems possible that the basis must consist in a commitment to the value of freedom, though in the absence of any direct argument for this conclusion, it must remain tentative.^24^ I foresee that the activity will take place; I must think that world containing the disapproved-of act will, *pro tanto*, be worse; and also that, nonetheless, there is reason not to stop it. Even if I had unrestricted power to implement my ideals it would still not be better to prevent the acts in question. I must restrain my powers of prevention in the name of letting these people get on with doing what they propose to do.^25^ In other words, the principle must involve granting the other person the freedom to act.

Counterfactual imaginability can even be applied to freedom, provided that someone’s being free is compatible with their always doing what I want. As I just noted, it is not clear why God could not have created a world in which people freely choose good over evil all the time (Mackie 1955) Alvin Plantinga and others attempt to show that though it is possible that a world exists in which people always freely choose to avoid evil, this is not a world which God could have created.^26^ This defence has in turn to ward off the objection that, given ‘middle knowledge’ (which Plantinga’s defence seems to demand) God could have confined himself to creating only those beings who would, at each point where they are faced with a choice between doing and avoiding evil, actualise the truth of a conditional that, if faced with such a choice, they will choose to avoid evil.

But, if granting it were *conditional* on their always ‘freely’ doing what I wanted, that would not count as an attitude which respected the other’s freedom. In other words, the freedom on whose basis the tolerator extends toleration must be one consistent with the lack of ‘middle’ knowledge – the knowledge of freely made choices – or with knowledge that the tolerated person will choose to do what she, the tolerator, believes to be wrong. My commitment to freedom must be such that I am prepared to accept that the other person will act in ways of which I disapprove and let them do so anyway. One basis for permission which resists counter–factual challenge is freedom.^27^

Looked at from another angle, there is an obvious reason why the power condition should have this effect. There is an inverse relation between power and freedom. If, for example in the circumstances of toleration, I use my power to prevent some act which you wish to perform, I have *prima facie* limited your freedom.^28^ If counterfactually I would use power I in fact lack to forestall your doing something of which I disapprove, I would limit your freedom, even if, things being as they are, I lack this power.^29^ The counter–factual here is used as a way of getting clear about my attitude here and now.

## The Wider Picture

Several conclusions may seem to follow. First, toleration has a dual linkage to freedom. On the one side, the tolerator must have the freedom to prevent the disapproved-of act which is conferred by having the power to do so. On the other side, the tolerated person must enjoy the freedom to perform the act. Hence toleration belongs in a structure of power which is, at least to some degree, dispersed among different agents, though of course this in no way demands an *equal* distribution of power. Second, toleration depends on attitudes of the agent. These attitudes are explicated dispositionally. It depends on dispositions whose truth is partly secured on that of counterfactuals. Since virtues are dispositions of a certain kind,^30^ the above account furnishes materials from which an account of toleration as a virtue may be constructed.

Third, the discussion suggests that justifications of toleration based on *modus vivendi* are faulty. According to such justifications, warring factions have good reason to tolerate one another when, as a matter of fact, none has the power to enforce its views on the other (s). So *modus vivendi* justifications deny that self-restraint can only count as tolerant when it rests on principle rather than on a pragmatic compromise: political deals reached because neither side enjoys a monopoly of power may nonetheless merit being called tolerant.^31^ As I have suggested, however, the way in which we describe or understand their actions here and now depends in part on how they would act counterfactually.^32^ No doubt *modus vivendi* is often a real political achievement. But the upshot is that the parties to it will not count as tolerant if they accept the arrangement only because they lack the power to get something better.

However, matters are less clear-cut than this. Politics has to operate with a notion of *embodied* freedom, rather than of freedom as mere indeterminacy. Political action always addresses a more or less limited menu of options, and this clearly places practical limits on idealisation. A regime of non-prevention may result because the limits on power mean that there is no bearable alternative to it; the idea that non-prevention through sheer lack of power was not toleration was, after all, what motivated the original power condition. Perhaps less obviously, it follows that toleration may be limited, not because of intolerant attitudes, but because political power itself is limited. It can be a real political question, whether something is tolerable – whether, that is, it *can* be tolerated.

The objections cut deeper. It is not easy to sustain any hard-and-fast line between ‘costs’ and ‘principles’. Political concerns typically straddle this line: for instance, does a government motivated by desire to secure public order, or to achieve civic peace without jeopardising other public services, act from a ‘pragmatic’ concern for costs, or from principle? This provides an illustration of the wider point that understanding in political theory is ill-served by a blank dichotomy between moral ‘principle’ and self-interested ‘prudence’. When political authorities act on behalf of the public interest, they typically take account of diverse interests and seek to arrive at an all-in decision about what to do. Political action is usually an amalgam of principle and prudence, telescoped in the notion of the public interest.

The further one goes in the direction of counterfactual challenge, the greater the risk of inconsequentiality. I suggest that the sole basis for toleration that could survive sustained counterfactual interrogation is a commitment to the tolerated party’s freedom. The point is not that nobody can ever wield enough power to forestall every action of which they disapprove. Nor is it only that the counterfactual’s remoteness disqualifies it as a basis for judging actual motivations, though it is certainly open to question whether claims as to how someone would act in the imagined situation shows how to evaluate their conduct here and now.

It is rather that there is no good reason to think that the counterfactual has the determinate truth-conditions it needs to do the job that theory assigns it. Suppose I had the supra-godly power that the imaginary case grants me; perhaps I would turn megalomaniac and not bother much about principles. It may be said that this introduces a gratuitous element into the fantasy. The intended response may be that, any rate, I *should* bother about the principles. But if the point is to justify a certain evaluation of my real-world acts, that will beg the question. The question is why one specification of the counterfactual is thought authoritative.

I propose the following formulation instead, which alternates but does not jettison PC_3_.

### Power Condition 4 (PC_4_)

A can only tolerate a disapproved-of action or state of affairs P if *either* (i) A believes that it is within his power to prevent (etc.) P and (ii) it is not the case that A would seek to prevent P, if A believed he had the power to do so, even if, in A’s judgement, prevention did not incur undue costs; *or* regardless of (i) and (ii) A accepts reasons for non-prevention over and above those of practical feasibility and cost.

It will often still be a real question what reasons A in fact accepts. That is one reason why indeterminacy is integral to political toleration. Given what was said earlier, it will also often be unclear whether the reasons for non-prevention make an act one of toleration. When confronted with the brute fact of a disapproved-of action, the putative tolerator may still think counterfactually that it would have been better that the action had not been performed, *and* that she would have prevented it, had she had the power to do so. This goes beyond the abstract reflection that a world without the action would be better, *pro tanto*, than one with it. The thought is that of an agent. I have argued that the only kind of reason for toleration which stands up to this counterfactual thinking is one which is based on commitment to the other party’s freedom, where the conditions of acting freely do not themselves necessarily include doing what the tolerator thinks is right.

Beyond this, however, lies a more basic contrast between those who accept toleration as a principled strategy in response to the reality of power – including the reality that everybody’s power is limited – and those who reject this. One version of the rejection lies in the attitude of those who merely restrain themselves, and accept a *modus vivendi*, because they lack sufficient power to enforce their will. Another is the ultra-idealism of theorists who imagine a world in which principles do not conflict, because there is, ultimately, only one principle. Such a stance is detectable in the work of some but not all liberal writers past and present.^33^ Even if it is accepted that the principles can conflict in theory, the ultra-idealist imagines that empirical conditions can be counterfactually reinterpreted so that the conflict fails to arise in practice.^34^

In general, ideals or principles themselves serve to frame what it is that I regard such a world as being. In projecting from this world to a more ideal one, I rely on principles. So, to retrieve an argument made earlier, the counterfactual test cannot be coherently applied to the principles themselves. Evidently, ideals themselves frame the content of the ideal world. We can appeal to a counterfactual judgement again, this time relating to what would be possible in one’s ideal world. One can coherently imagine away many unpalatable realities in one’s ideal world. For example, I can imagine that I have more power, in the form of greater resources, physical capacities, etc., than I in fact have. But principles cannot coherently enter into what I can counterfactually imagine in the same way: I cannot usually imagine that the principles I hold in the world as it is, are ones which I would not hold in an ideal world. There is the case mentioned earlier of someone who holds principles that he thinks he would not hold in an ideal world. But that case is clearly marginal. Someone who thinks that must already have gone some way towards jettisoning the supposedly real-world principles in favour of the supposedly ideal ones. The point in general stands, that idealisation is relativised in general to the ideals or principles one actually holds.

Unlike costs or the material resources needed to wield greater power, I cannot coherently see my purely principled incapacity to do something as cause for regret, from which I would not suffer in some other and more ideal world. We can make clear the contrast between costs of principle and other costs by considering a case in which the agents are restrained only by a pseudo-principle of efficiency, as in the Nazi example given earlier. But it seems clear that the notion of efficiency here does not have the standing of a principle.

Ideals or principles one holds serve to constrain what one can do. They are not subject to revision through the counterfactual idealisation that motivations the power conditions, since they frame the content of that idealisation. This prompts the broadly anti-Kantian thought that the ideal of freedom at which the counterfactual test arrives is too thin to gain much political traction. This is not because an abstract ideal of freedom is politically irrelevant: indeed, it helps to make politics what it is. But that ideal requires a normatively thickened account of circumstantial freedom, to which appeal is made in (for example) judgements about whether a policy or act is tolerant. That a decision – for instance, about whether to tolerate some practice or group – is political stems from its relying on a specific notion of freedom, even while the abstract notion remains in the background as a challenge to it. Its being political requires, but at the same time cannot rest at, the idea of freedom as simple indeterminacy.

In fact, the emphasis on radically contrary-to-fact possibilities also ignores the resources afforded by a more modest form of idealisation or generalisation, which deliberates from extant commitments or values. One form that can take is to ask how far an agent’s actions are those of a tolerant person – that is, someone with the virtue of toleration.^35^ Once we abandon the idea that describing an act as ‘tolerant’ must commend it, there is less room for puzzlement that the act of coexisting in conditions of mutual attrition may embody a certain form of self-restraint, without expressing tolerance as a virtue. That is true – and this was the initial plausibility in the power condition and the underlying notion of an ideal world – of those who settle on peace or *modus vivendi* while heartily wishing that the other side did not exist, would convert, or go away. Lack of power may also impose limits both on what sort of agents the parties to a *modus vivendi* can be, and on what normative descriptions of them and their actions are appropriate.

The radically counterfactual notion of freedom explored above with regard to those tolerated has its counterpart in a similarly radical understanding of the tolerator’s freedom. This surfaced earlier, in the idea that the tolerator might renounce even the principles by which counterfactual ideals are framed. Perhaps, one may say, the tolerator’s evaluative dispositions could and should have been different. Here again, the problem is not in filling out the pertinent counterfactual ‘in principle’. It is that there is no good reason in general to think that a shift from this world to the relevant counterfactual one lies within the tolerator’s power. Ethical dispositions, including evaluative ones, are not the sort of thing that can be put on and taken off like a coat: it is plausible to think that their not being like this helps to make them ethical dispositions.

The picture I am resisting is of pared-down notion of agency in which the supposed toleration turns out to be a misnomer, on the grounds that there is some counterfactual world where the tolerator lacks the ethical dispositions that she in fact has. The dispositions themselves constrain the power the agent has. The imputation is that because it is conceivable in principle that the tolerator might have been free of the dispositions engaged in toleration, she should not be accounted tolerant. If however it is right to say that it does not lie within her power to slough the dispositions off, this is not something she *can* do. But for reasons already given, the situation is very different from one where someone signs an edict of toleration at the point of a gun: the lack of power to do otherwise does not efface toleration.

What remains contestable is the notion of freedom on which such judgements rest. That can be contested politically as well as philosophically. There is nothing to rule out the pared-down notion of freedom being traded in political argument; but there is also nothing that gives it special authority. In that sense, the radically thinned-out notion of freedom as conformity to a supreme principle may be thought to embody not the moral apotheosis of toleration, but a *reductio* of it.

## Conclusion

In sum, the tension between the power condition and *modus vivendi* toleration remains. But it can be extended to other more ‘principled’ cases of putative toleration where the tolerator’s stance is vulnerable to counterfactual idealisation. Even so, the temptation to achieve clarity through such idealising devices should be met with caution. The main reason for this, in my view, is that conceivability casts its net wider than deliberative possibility. What lies within one’s power, at least at the level of conscious action, depends on what is deliberable, which is subject to character-based constraint. While external curbs, such as a rough balance of power, may limit what can be done anyway, people in these circumstances can still entertain different ways of thinking and acting in response to it. On a dynamic rather than static view of the situation, a pragmatic truce may evolve into something beyond that, while being sanctioned within the set of values that those involved actually rather than ideally would hold.

This paper has probed the question of what is fixed and what is variable in thinking about toleration. On the view advanced here, toleration is located somewhere between pure power-play and pure idealism. The space for toleration is thus limited, but not vanishingly small. Toleration is not in the place where we ideally would like it to be, since that is fixed by the very principles which toleration qualifies. As such it is uncomfortable and often unstable. This makes it doubly unwelcome to ideal theory. The ironic movement traced by the argument of this paper is that as one tries to strain and refine toleration, by ridding it of the impurities deposited by political reality, they crop up again as aporias of theory. On this point, at least, John Horton would I suspect concur.

It is hard to stand toleration. It proceeds from accepting that the sub-ideal is given as such – though the form it takes may not be – and we have to do the best we can with it. That means accepting the fact of power as an ineradicable feature of political life, even while acknowledging the limits on what we have the power to do, in theory as in practice.

## Footnotes

1 See Horton (1994)

2 ‘Why the Traditional Conception of Toleration Still Matters’(Edyvane and Matravers 2011)

3 It is partly in view of this that John Rawls regarded *modus vivendi* accommodations as ‘mere’ compromises which were ‘political in the wrong way’ and hence lack the virtue of stability. See Rawls (1995)

4 This qualification is not redundant. One’s capacities depend not only on skills, knowledge, etc., but also on whether, in the circumstances, one can exercise these skills.

5 Cf. the old teaser: *Could God create a stone so heavy that he could not lift it?*

6 E.g. Cohen 2004; Scanlon 2003; 1998

7 Cf. Forst 2003; Jones 2007.

8 Forst’s term: *Toleranz im Konflikt*, e.g. 127.

9 An important question concerns the *relevant counterfactual set*, the truth-conditions of whose members bear on the truth of the judgement that A tolerates P. What counterfactually varies is the power A exercises. But sometimes the fact that A has additional power alters the practical environment for others (the putatively tolerated), and with it the relevant counterfactual set. A’s greater power may work proleptically, deterring people from doing P, so no question of toleration arises.

10 For this reason, on my analysis it is sometimes indeterminate whether someone tolerates a practice.

11 Political principle itself, such as commitment to an ideal of reciprocity, is also often based on self-interest.

12 The further claim may be made that instituting a *modus vivendi* now may encourage the formation of genuinely tolerant attitudes over time.

13 See Horton and Baumeister (1996)

14 Horton proposes a solution along the lines mentioned and criticised in the text. For further discussion see also *Virtue, Reason and Toleration*, ch. 4.

15 For an example of this process, see Cohen 2002

16 This case is also different from one where I imagine that it is better that my principles are not generally held. I could think that the world is better for the fact that *I* hold them, although it’s also better that others don’t.

17 It may be said that if I see my principled beliefs as formed through brainwashing, etc., I to that extent don’t hold the principle at all. I may believe that children may be subjected to corporal punishment, but also think that this belief is one I hold only because of my own peculiar upbringing. But if I regularly flog my offspring despite holding this meta-belief, I still act on the belief that flogging them is appropriate.

18 In the United Kingdom, for instance, the Terrorism Act 2006 extends the period for which suspects may be detained without charge to 28 days.

19 See Rawls 1971

20 See Marcuse 1969; Brown 2008

21 I owe this example to Graham Long.

22 One version of this thought is the ‘dominant ideology’ critique of liberal capitalism. However, for the agent to think that this vitiates his own toleration would, on this critique, require that he has unmasked his own ideological commitments, i.e. has become, in a manner of speaking, a critical theorist.

23 See for example Geuss 2008; Stears 2007

24 It might be said that other considerations could yield this response, such as belief in the finitude of human knowledge or reason (anonymous reader’s comment). But it seems that this kind of incapacity can still be subject to (of course strongly counterfactual) idealisation.

25 It is far from clear that I myself cannot figure among the doers. If I can, I can tolerate myself. That is, I can act in ways of which I disapprove (*pro tanto*) in the name of bringing about a morally maximal world.

26 Or, more weakly, God might not have created such a world, so it is possible that God might not have created a world which both contains free will and from which evil actions are absent. See Plantinga 2002

27 It may be that an executive virtue such as integrity also remains immune to the counterfactual challenge.

28 Only *prima facie*, because your capacity so to limit me may be something to which I have freely assented. See Williams 2005

29 If your merely *prima facie* freedom would be counterfactually limited, it can be argued that I act tolerantly, since I have warrant sanctioned by you for limiting your freedom in those circumstances.

30 Aristotle, *Nicomachean Ethics*, 1105b25ff.

31 See Gray 2000a, b.

32 On some views, these counterfactuals may lack determinate truth-conditions. If so, so may the corresponding judgements about whether A acts tolerantly in failing to stop P.

33 Isaiah Berlin and Ronald Dworkin respectively exemplify the many- and single-principle forms of liberalism. See Gray 2000a, b.

34 This stance can also be seen in apparently less extreme forms of idealism, where principles themselves are given, and come into conflict, but theorists devise strategies for eliminating conflict. The doctrine of double effect constitutes one casuistical strategy along these lines.

35 I sketch an account along these lines using Aristotelian materials in *Virtue, Reason and Toleration*, ch. 3.
